# Expectations and perceptions of primary healthcare professionals regarding their own continuous education in Catalonia (Spain): a qualitative study

**DOI:** 10.1186/s12909-017-1061-6

**Published:** 2017-11-15

**Authors:** Xavier Mundet-Tuduri, Ramon Crespo, Ma. Luisa Fernandez-Coll, Montserrat Saumell, Flor Millan-Mata, Àngels Cardona, Núria Codern-Bové

**Affiliations:** 1 Training and Research Unit of Primary Care in Barcelona city. Catalan Health Institute, Universitat Autonoma de Barcelona , Bellaterra, Spain; 2 Institut Universitari d’Investigació en Atencio Primaria Jordi Gol. (IDIAP Jordi Gol), Postal Address: C) Sardenya 375, 08025 Barcelona, Spain; 3 ÀreaQ. Evaluation and Qualitative Research, Barcelona, Spain; 4 Training and Research Unit of Primary Care in Barcelona city, Catalan Health Institute, Barcelona, Spain; 5 Escola Universitària d’Infermeria i Teràpia Ocupacional de Terrassa (EUIT), Universitat Autónoma de Barcelona, Bellaterra, Spain

**Keywords:** Primary care, Continuing medical education, General practice

## Abstract

**Background:**

The planning and execution of continuous education in an organization that provides health services is a complex process. The objectives, learning sequences, and implementation strategies should all be oriented to improving the health of the population. The aim of this study was to analyse the expectations and perceptions of continuous educations by primary healthcare professionals (physicians and nurses) and identify aspects that hinder or encourage the process.

**Methods:**

A qualitative study with 5 focus groups made up of 25 primary healthcare professionals from the Catalan Health Institute, Barcelona (Catalonia, Spain). The focus groups were audio-recorded and the results transcribed. The analysis involved: a) Reading of the data looking for meanings b) Coding of the data by themes and extracting categories c) Reviewing and refining codes and categories d) Reconstruction of the data providing an explanatory framework for the meanings e) Discussion about the interpretations of the findings and f) Discussed with relevant professionals from PHC (physicians and nurses)“Data regarding thematic content were analyzed with the support of Atlasti 5.1 software.

**Results:**

The health needs of the population were often at the core of the learning processes but the participants’ views did not always spontaneously refer to improvements in these issues. Common themes that could hinder learning and where identified, including contextual aspects such as work constraints (timetables, places being covered during training) and funding policies. New learning strategies to improve the effectiveness of continuous education were proposed such as the exchange of knowledge, the activation of personal commitment to change, and the improvement of organizational aspects.

**Conclusions:**

The primary healthcare professionals in our study viewed continuous education as a professional necessity and would like to translate the knowledge acquired to improving the health of the population. Nevertheless, professional, structural, and organizational issues impede the process.

## Background

Today, continuous education (CE) for physicians (continuous medical education or continuous professional development [CPD]) and nurses (continuous nursing education [CNE]) working in primary healthcare is essential to gain more knowledge and skills, with the aim of improving the health of their patients and the population in general [1–5].

**Fig. 1:**
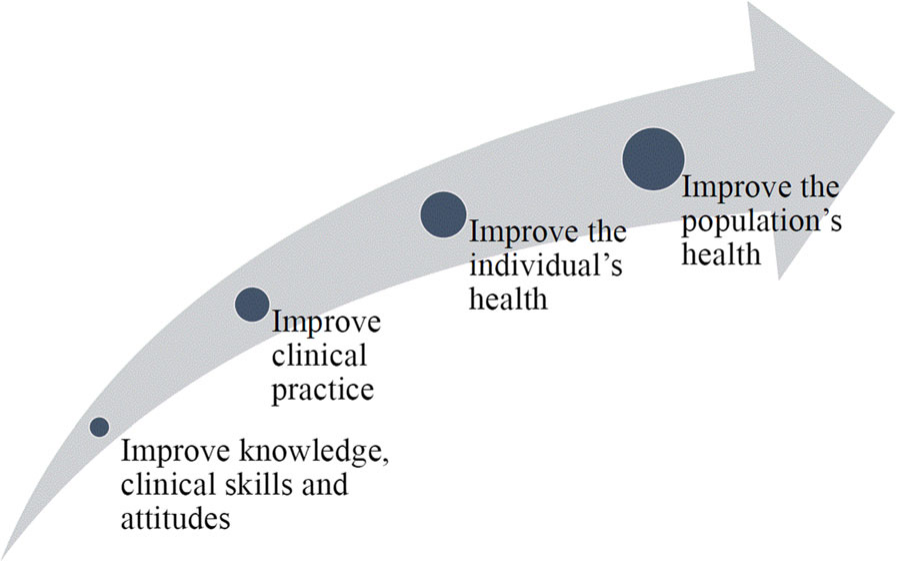
Objectives that should be included in continuous training

CPD and CNE objectives should respond to the health needs of the community. By improving professionals’ knowledge, clinical skills, and attitudes, we enhance clinical practice and, consequently, patient and community health (Fig. 1).

According to available evidence [6], educational interventions should be relevant and intense (so as not to waste time or reduce that dedicated to patient duties), be change-oriented, and interactive (engage the participants and encourage their involvement). Finally, it should motivate healthcare professionals to change their attitudes and behaviour [7, 8].

All of the above makes up the conceptual framework that is the CE basis for the research team of this article.

Moreover, it is important that the interventions should take into account the various stages that make up the learning process. The professionals should understand the aim of the training activity, the impact on the clinical practice, and the expected results for the population’s health [2, 9, 10]. A good method to achieve this is to explain ‘how’ and ‘to what extent’ each educational activity is related to the three elements in the learning process. They are a) Predisposing factors: those that create awareness about a specific or communal need. b) Activating ones: those that help improve clinical practice by generating knowledge, skills, attitudes and behaviour. And c) Reinforcing ones: those that encourage the sustainability of the improvements obtained and help to overcome the barriers that the system may place at the moment of implementing what has been learned [2, 11].

Sharing this information (detailing whether it is an activity aimed at predisposing the participants to change, enabling them to make changes in practice, or reinforcing improvements) is what is known as ‘visible pedagogy’ [12]. Applying this methodology can facilitate learning and aid in overcoming barriers to change [12].

Finally, CE should be designed from a strategic perspective [5] and incorporate issues from the professionals’ environment that encourage or hinder their educational activities [13]. The issues that the literature most often refers to include: the characteristics of the professionals receiving the training, the organizational system of the primary healthcare.

The study was conducted in Catalonia, a Mediterranean region in northeastern Spain. Catalonia has a public health system in which primary care is organized into primary health centers (PHC). Each citizen is registered with an individual general practitioner (GP) and a nurse in one of these centers. The main health provider in the region is the Catalan Health Institute, a publicly funded health care system that operates 279 health care centers attending 5.8 million patients (80% of the region’s population). In every PHC there are one or more professionals, known as ‘physician and/or nurse CE leader’, who plan and organize CE based on the perceived needs of the team. Depending on the content of the course, the GPs and nurses may attend CE together or separately, at their own health centers, within working hours. The professionals (GPs and nurses) also attend voluntary training activities financed by the Catalan Health Institute and centrally organized by the Training Unit. As a result, PHC professionals annually receive from 3 to 5 courses/workshops related to CE in their own health centers and, additionally, 3 to 4 other courses from the more than 200 ones offered by the Catalan Health Institute. CE is voluntary, nevertheless, the Catalan Health Service asks for a minimum number of annual credits in order to obtain professional accreditation and the corresponding salary increase. The CE organized by the Catalan Health Service is free or the participants, whereas any CE that is not included must be paid for.

Every year the Training Unit of the Catalan Health Institute carries out a survey to identify the training needs of the PHC professionals. It is concerned about the efficacy of CE and the manner in which research findings are incorporated into the educational activities so that the population’s health can be improved. Moreover, the way the PHC professionals view CPD and CNE appears to be relevant in order to obtain better results from the training program [14]. Such an issue can affect both decision- making regarding the training activities to be performed and their design.

The first step to improving CE efficacy is based on understanding the viewpoints of the PHC professionals, and the context in which they register for courses [15, 16]. Prior to incorporating any modifications to the development of effective CE, a qualitative study should be performed that identify and analyse the significance of CE for PHC professionals and how they think it can improve the population’s health. This study should also aim to establish aspects, both of planning and context, that aid or hinder the learning process.

At present, it is unclear whether the PHC professionals in Catalonia incorporate the conceptual framework of CE into the design of their training activities. Each professional is responsible for their own policy. For this reason, there is a lack of data regarding acceptance (or not) and the reason why (or why not).

The organization of primary care and the CE of its professionals in the rest of Spain is very similar to that of Catalonia. As a result, although our study was carried out in Barcelona, we believe that our findings can be extrapolated to other areas in the country of Spain, as well as other countries that may have a similar healthcare system and CE program for their primary healthcare professionals.

## Methods

### Aim

The aim of this study was to analyse the expectations and perceptions of continuous educations by primary healthcare professionals (physicians and nurses) and identify aspects that hinder or encourage the process.

### Design of the study

This qualitative study was performed using focus groups (FGs) composed of PHC professionals [17]. The research team was made up of experts in qualitative evaluation and research (ÀreaQ) and professionals from the Training Unit for the city of Barcelona from the Catalan Health Institute. They have more than eight years of experience in the planning and evaluation of CE for the largest supplier of health services in Barcelona (52 primary health care centers and 2674 health care professionals).

### Setting

The study was carried out in Barcelona (Catalonia, Spain) during 2014.

### Participants

The study participants were PHC professionals (GP and nurses) working in Barcelona who had previously used the CE provided by the Catalan Health Institute. Participant selection was carried out by a purposeful sampling method [18]; this a method whereby participants are selected based on characteristics that will reliably inform the research question. The potential eligible participants were stratified by the following criteria, so that there was maximum variation in the participant characteristics [19], providing the maximum possible representative sample: age (≤49 years, ≥50 years), gender, type of profession (physician/nurse/CE physician leader in PHC centre/CE nurse leader in PHC centre), labour status (permanent/interim staff), and type of PHC centre (teaching centre or not).

Recruitment of participants for the FGs was carried out by the research team. An e-mail was sent to 87 PHC professionals (GPs or nurses) informing them of the objectives of the study and asking for collaboration. Later, they were called and 35 agreed to take part in the study. Finally, 25 participated (Table 1). In all cases, non-participation was due to incompatibility with work schedules.

**Table 1:** Social and laboral characteristics of the focus group participants

Variables	Focus groups					TOTAL
	GF1	GF2	GF3	GF4	GF5	
Age						
≤ 50 years	3	1	3	4	1	12
>50 years	3	2	2	3	3	13
Gender						
Female	3	1	4	7	4	19
Male	3	2	1	0	0	6
Type of profession:						
Nurse	0	0	0	7	4	11
Physician	0	3	5	0	0	8
Nurse leader of CE in PHC centre	3	0	0	0	0	3
Physician leader of CE in PHC centre	3	0	0	0	0	3
Employment situation						
Permanent staff	5	2	3	6	3	19
Interim staff	1	1	2	1	1	6
Type of primary health team						
Teaching centre	2	2	3	3	2	12
Non-teaching centre	4	1	2	4	2	13

### Data collection

FGs were considered the most suitable technique to gather data as they allow the researcher to generate a discussion both with and amongst the participants about the issues of interest [20]. Five FGs were carried out in a space not located in the work centers. The characteristics of the FG are described in Table 1. They were conducted for an average of 90 min at midday to encourage participation of both morning and afternoon shifts. The FGs were conducted by a member of the research team with experience of moderating FGs, who followed a thematic guide that had been previously developed by the research team, but which could be altered during the course of the session (Table 2). An observer was also present and was taking notes on the FG discussions.

**Table 2:** Thematic guide for the focus groups

1. - Commencement: Presentation, summarized explanation of the study. Conditions of participation and signed informed consent. 2. - Introductory question: From your own experience, if you had to explain to someone what continuous training was, what would you say? 3. - Opinions regarding the expectations of continuous training. a. Discover how the ideal training plan should be formed (criteria, results). b. Needs related to continuous training. c. Skills to be achieved through continuous training (soft and hard ones) and their relationship to improved health care. 4. - Opinions regarding the use of continuous training. d. Discover how to adapt the continuous training plan to ones expectations and needs. e. Aspects that encourage and hinder continuous training (timetable, location, quality teaching content, current working context). 5. - Opinions regarding the health system and continuous training. f. Drafting the training plan. g. The role of the continuous training managers. h. Learning methodology. 6. - Proposals for improvement. 7. - Closure: farewell and thanks to the participants.	

Both the driver and the focal group observer were two research team researchers unconnected to the EC of the Catalan Health Institute. The researchers did not know the members of the groups, just as the participants did not know each other. In this way, the professionals felt free to express their ideas and opinions.

### Type of analysis

The FG were audio-recorded and the results transcribed. A content analysis [20] was carried out from a socioconstructivist approach [17], since it was understood that the attitudes and behaviours with respect to CE were, largely, social constructions. The analysis involved identifying the explicit and manifest content of the participants, grouping into thematic categories the different elements that emerged. In addition, it examined the explicit relationships amongst them following a coding system based on the conceptual framework for CE (see Background) [20].

The aim of the analysis was to examine the discourses from the transcribed results and provide meaningful interpretations of the FGs participant discourses. The conclusions and recommendations were made looking for patterns or consistent themes from the different participants of the FGs. [21].

The conceptual framework for continuing education (see background) was used for the identification of categories and interpretation of data.

The analysis involved the following procedures: a) Reading and re-reading of the data looking for meanings, and writing down the first intuitive pre-analytical reflections (NCB and RC independently); b) Coding of the data by themes and extracting categories according to research questions and the conceptual framework for continuing education (NCB and RC independently); c) Reviewing and refining codes and categories (NCB and RC); d) Reconstruction of the data providing an explanatory framework for the meanings of the different views (NCB and RC); e) Discussion about the interpretations of the findings with the whole research team (NCB, RC, ML FC, MS, FM AC and XMT) to insure its plausibility and understand ability and f) As a final step, to increase the credibility of the study, the results were presented and discussed with relevant 80 professionals from PHC (physicians and nurses) working in the city of Barcelona.

Thematic data analysis was supported by Atlas-ti 5.1 software.

## Results

The following themes emerged: a) Defining CE, b) CE planning: detection of training needs, c) educational strategies, and d) CE contextual aspects.

- Defining CE

The FG viewes revealed that whilst the population’s health needs were frequently placed at the core of the learning processes, it was also true that ‘improvements in the population’s health’ were not explicitly included as a reference indicator when determining the quality of an educational strategy. CE is usually considered ‘good’ only when the PHC professionals feel satisfied afterwards and the acquired knowledge can be easily transferred at an individual level.

*“to evaluate the CE I would say satisfaction and up to what point it influences daily practice … and to what point it modifies your practice” (P1:GD3)*.

In this regard, the participants established a parallelism between CE and ‘good professional practice’ so that ‘doing CE’ was defined as a strategy to guarantee quality care for individuals.

*“I would like to get this skill as soon as possible so that I can work calmly … if I don’t get the necessary knowledge to work with these two complex pathologies … This makes me feel unsure in my work and uncomfortable” (P3:GD5).*

Such a strategy includes the renewing, from a holistic and integral perspective, of specific skills, including transversal (core) ones (i.e. interpersonal, technological and specific professional skills, critical analysis), all of which are considered necessary for an up-to-date, high quality, everyday professional activity.

“*Training must be focused on what will be really useful in practice and include updating, basically updating”* (P4:GD5).

“*A methodology that helps us evaluate in a continuous way our practice, reflection about difficult situations”* (P3: GD4).

Some PHC professionals interpreted CE as being more than just the acquisition of regulated skills and linked it to other kinds of results. From this analytical point of view four important considerations were obtained. The first was that CE was seen as a company strategy to obtain specific goals for the organization. Habitually, when referring to this kind of objectives, CE solely concerned issues related to quality, internal coordination, or the introduction of new protocols and procedures.

*“The company establishes some objectives, then ... the difference between what you already know and what the work center asks for, the difference of knowledge is, is what you have to obtain through continuous training” (P3:GD5)*

An explicit link was sometimes identified between the strategic component of the CE and a systematic approach to the population’s health problems.

*“Before we had a population with bed sores, protocols and training were given and now there are hardly any” (P2:GD1)*

Another point concerned CE and how it could lead to an improvement in the professional’s career:

*“A lot of people take courses because they are good for their careers, not because the course itself is interesting [...] they have said to me “I’m taking this course, not because it’s interesting but I know they’ll ask me to do it.”(P4:GD3)*

The potential of CE to motivate professionals, improve teamwork, and prevent burnout situations was also acknowledged. This view predominated among groups composed by CE physicians and/or nurse’s leaders.

*“CE is what gives you better services, skills, and it is a benefit for them, it motivates” (P3:GD1)*

*“There are courses that are necessary to de-stress, unblock, to relax. Every Friday we have a session of this type and now we even pay for it ourselves” (P1:GD1)*

Finally, CE was related to covering personal learning interests not directly linked to daily professional practice.

*“This is my point of view: to cover necessities. If (any time) is left over I try to grow as a professional”* (P2:GD4).

b) CE planning: Detecting training needs

The analysis revealed the importance of identifying training needs based on the health needs of the population. Nevertheless, the participants often showed difficulties in describing how they were incorporated, in a systemized manner, into training requirements.

Two interesting matrices can be drawn from these views. On the one hand, the current circumstances of the economic crisis, and the resulting cuts in funding for the healthcare organization, do not favor an environment for the comprehensive identification of training needs. That is to say, in spite of the efforts made to identify training requirements, it is not always possible to completely involve the professionals in determining their training needs: it is limited to asking for their preferences, analyzing deficits, and incorporating needs for both professionals and patients. CE leaders at the PHC center said:

*“We carried out a questionnaire with respect to concerns and there was very little response. If there is not much response you offer training and ask if they’re interested” (P2:GD1)*.

*“We received a course on how to make a continuous training plan. We gave out questionnaires about training needs and also received few responses. As a result, I ask the team directly. Finally, the courses were made with common sense criteria, the company’s strategic lines, developments and what people said” (P5:GD1)*.

On the other hand, the offer of CE activities does not always coincide with what the professionals consider a relevant proposition.

*“Three courses have to be chosen out of all the possible ones and they are always full because they are the most interesting and so later there aren’t any places or only for the people in charge. All the rest (...) it leaves a lot to be desired” (P2:GD2)*

c) Educational strategies

With regard to what is known as CE interaction (understood here as the capacity of educational interventions to connect new concepts, ideas, and skills with the participants’ prior experience and knowledge), it can be seen that a considerable number of the contributions referred to the value given by the professionals to educational strategies which imply the adaptation of CE to the participants’ background.

*“When we have psychoeducational groups we learn because they carry out the supervision for us, we share space, you comment and learn. Or in the hospitals, where we share cases. Sharing cases helps us learn things about how to approach them or carry out specific consultations”. (P2:GD4)*

In this regard, the marked dislike expressed by the PHC professionals with regard to strategies that do not follow this logic (for example, theoretical classes not related to practice) leads to the conclusion that this is an issue which deserves to play a key role in the pedagogical policy encouraged by organizations responsible for CE planning. Moreover, the professionals described new forms of learning which promote, amongst other things, the exchange of knowledge and proficiencies. This approach i.e. the interactive exchange of ideas and knowledge, could therefore be incorporated into the design or redesign of (new) courses at the centers. In this respect, an additional question considers the notion that educational interventions should have more informed individuals participating. Contributions regarding this issue lead to the idea that, for the PHC professionals, it is important to place the educational proposals they are offered within the broadest framework of a pedagogical strategy aimed at improving the population’s health.

*“I feel I miss what is being said, CE that will help me grow as a professional in the sense of being critical about the daily work I do, which functions a bit like feedback, day by day”* (P3:GD4).

Nevertheless, at the same time difficulties have been observed when identifying the strategy or the real sense of the educational proposals provided and, as a result, the problems of relating them to the learning process.

*“I don’t want to be trained in order to fulfill objectives”* (P3:GD5).

Another key aspect in CE effectiveness is motivating the professionals to employ in their daily practice the knowledge they have obtained and thus improve the population’s health. Our study demonstrates that whilst this issue is absent in the professionals opinions, this does not imply that there is no commitment to change, but rather that it not explicitly manifested.

d) Contextual aspect of CE

The PHC professionals also mentioned the challenges of everyday working practice affecting CE. These aspects concern difficulties, such as pressure to attend work which hinders being present at training, the availability of information about the courses, the accessibility to places and times, the quality of the trainer and/or the role of the Catalan Health Service (particularly in relation to the absence of CE accreditation for the professionals and lack of funding due to budget cuts).

*“The problem with the courses provided on the web is the number of places, we are a lot of people and there are 5 places” (P3:GD3)*.

*“As a professional you need training and you don’t have time or know how to do it, you do it with time from your personal life” (P2: DG2)*.

In addition to these issues, there were also contextual aspects that had improved over the last years. There were no differences amongst the various members of the FGs about this issue.

*“For a year and a half we have had courses provided on the web. It’s fantastic and you have all the training more or less all year” (P1: GD5)*.

## Discussion

This study describe the views of the PHC professionals with respect to CE, including the difficulties and opportunities that it offers.

The first result is that participants perceive CE as a tool that improves individual professional care but they did not spontaneously comment on it being a strategy directly linked to enhancing the health of the population.

These findings are consistent with a systematic review by the EC. The scarcity of EC conceptual frameworks with the aim of interpreting the needs of the population and designing strategies to improve the health of the population and vulnerable groups [22].

The literature reports that one of the limitations of a traditional CE focus is that it is often directed at changes at the level of individual behavior. This attitude hinders the transfer of knowledge to professional practice [2, 14].

Secondly, and with regard to aspects that could hinder the learning process, there were slight variations in opinions amongst the groups. Some of the concerns expressed to the CE leaders are limitations in identifying training needs and the lack of a policy of professional accreditation within the framework of primary care. In concurrence with Davis & Davis [2], such aspects play a key role in CE. It is therefore proposed that a number of strategies be incorporated in order to identify needs, including standardized evaluations of the knowledge, skills, and observations of the professionals’ practice. Various researchers have experimented with questionnaires based on medical practice [23, 24] and taxonomies of clinical practice [25], and valuable results have been obtained for the improvement of CE. Others difficulties encountered by the PHC professionals are the limitations in the information offered by the organization about the characteristics and the finality of the educational intervention. All of the participants referred to common themes impeding learning, which were related to contextual aspects such as labor constraints (timetables, their workplace being covered while they were attending training sessions), and funding policies. These aspects are in agreement with the barriers to learning described in the literature [15, 26].

Thirdly, the elements that favored CE were: a) coordination amongst the various levels of the health system; b) the empowerment provided by systematic secondment in hospital services; c) technological reinforcement (for example, online courses, information about these courses, online group discussions) in order to improve accessibility to CE; and d) the use of small groups to reinforce learning. The literature recommend combin these strategies to augmenting CE efficacy [2]. García García et al. [27] have reported increased opportunities in terms of CE through the coordination between primary and specialized care. The improvements being obtained in the referral process for patients to nephrology external consultancy services. Nevertheless, a recent systematic review [28] revealed that research is still limited with respect to the efficacy of multiple strategies to improve complex health issues.

We compare our study with other ones carried out in countries like Canada and Sweden [29, 30] that have a primary care system similar to Catalonia. Professionals have similar barriers. They propose a CE adapted to the daily life of professional and interprofessional education in order to learn to work in teams. Secondly, the need to use pedagogical methods that allow a high level of reflection and understanding of the subject to be treated [9].

Our work has strengths and limitations. The use of a qualitative approach and focus groups is a strength. It allows to us in depth knowledge about the difficulties and opportunities that CE offers our PHC professionals. This information is indispensable for the improvement of the CE programs in the future [14]. As a limitation, it should be noted that not all the focus group member profiles were homogeneous, as a result, there was a high variability of opinions so that the problem was reflected with amplitude.

The findings from this study prompt research to establish if planned CE, adopting this conceptual framework, improves the health care of the population served.

## Conclusions

PHC professionals (GPs and nurses) consider CE as a necessary learning process: not only for their personal growth but also (and more importantly) as something for improving the health of the population. The professionals, however, detect difficulties in identifying the health needs of the populations. As a result, the CE programs (courses or workshops) are not always focused on responding to the population’s necessities.

Secondly, any CE activity not based on sharing experiences and expertise from the perspective of practice loses value for the PHC professionals (GPs and nurses).

The study findings suggest the following recommendations that may be advisable to implement in CE in the PHC team:

- The CE proposals should explain how the educational activity will result in improved professional skills leading to better professional practice and, as a consequence, enhanced health of the individual and the population.
- The CE plan should be drawn up by the PHC team so that it promotes change-oriented behaviour, taking into account that there are certain activities that predispose, activate, and encourage the PHC professional to change.
- It is important that the detection phase of the training needs includes, in a systematic manner, those of the population.
- CE activities employing interactive strategies that strengthen collaborative learning should be encouraged. In addition, the use of educational strategies based on practice, such as case analysis, should be promoted.

## Abbreviations

CE: Continuous education
CNE: Continuous nurse education
CPD: Continuous professional development
FG: Focus group
PHC: Primary health center

## References

[1] Curran JA, Grimshaw JM, Hayden JA, Campbell B (2011). Knowledge translation research: the science of moving research into policy and practice. J Contin Educ Heal Prof.

[2] Davis D, Davis N (2010). Selecting educational interventions for knowledge translation. CMAJ.

[3] Leape LL, Berwick DM (2005). Five years after to err is human: what have we learned?. JAMA.

[4] McGlynn EA, Asch SM, Adams J, Keesey J, Hicks J, DeCristofaro A (2003). The quality of health care delivered to adults in the United States. N Engl J Med.

[5] Moore DE, Green JS, Gallis HA (2009). Achieving desired results and improved outcomes: integrating planning and assessment throughout learning activities. J Contin Educ Heal Prof.

[6] Grimshaw JM, Eccles MP, Lavis JN, Hill SJ, Squires JE (2012). Knowledge translation of research findings. Implement Sci.

[7] Hoof TJV, Meehan TP (2012). Using theory and evidence to guide the use of educational outreach to improve patient care. Am J Med Qual.

[8] Øvretveit JA (2004). Framework for quality improvement translation: understanding the conditionality of interventions. Jt Comm J Qual Patient Saf.

[9] Forsetlund L, Bjørndal A, Rashidian A, Jamtvedt G, O’Brien MA, Wolf F, et al. Continuing education meetings and workshops: effects on professional practice and health care outcomes. Cochrane Database Syst Rev. 2009:CD003030. 10.1002/14651858.CD003030.pub2 PMC7138253 19370580

[10] Timmings C, Khan S, Moore JE, Marquez C, Pyka K, Straus SE (2016). Ready, set, change! Development and usability testing of an online readiness for change decision support tool for healthcare organizations. BMC Med Inform Decis Mak.

[11] Best A, Stokols D, Green LW, Leischow S, Holmes B, Buchholz K (2003). An integrative framework for community partnering to translate theory into effective health promotion strategy. Am J Health Promot.

[12] Bourne J, Muller J, Davies B, Morais A (2004). Framing talk: towards a ‘radical visible pedagogy’. Reading Bernstein, researching Bernstein.

[13] Mazmanian PE, Davis DA, Galbraith R (2009). American College of Chest Physicians Health and Science Policy Committee. Continuing medical education effect on clinical outcomes: effectiveness of continuing medical education: American college of chest physicians evidence based educational guidelines. Chest.

[14] Kitto SC, Bell M, Goldman J, Peller J, Silver I, Sargeant J (2013). (Mis)perceptions of continuing education: insights from knowledge translation, quality improvement, and patient safety leaders. J Contin Educ Heal Prof.

[15] Baxter P, DiCenso A, Donald F, Martin Misener R, Opsteen J, Chambers T (2013). Continuing education for primary health care nurse practitioners in Ontario, Canada. Nurse Educ Today.

[16] Clark E, Draper J, Rogers J (2015). Illuminating the process: enhancing the impact of continuing professional education on practice. Nurse Educ Today.

[17] Denzin NK, Lincoln YS (2011). The SAGE handbook of qualitative research.

[18] Crouch M, McKenzie H (2006). The logic of small samples in interview-based qualitative. Research. Soc Sci Inf.

[19] Teddlie C, Yu F (2007). Mixed methods sampling: a typology with examples. J Mix Methods Res.

[20] Braun V, Clarke V (2006). Using thematic analysis in psychology. Qual Res Psychol.

[21] Kitzinger J, Barbour R (1999). Developing focus group research: politics, theory and practice.

[22] Dawson AJ, Nkowane AM, Whelan A (2015). Approaches to improving the contribution of the nursing and midwifery workforce to increasing universal access to primary health care for vulnerable populations: a systematic review. Hum Resour Health.

[23] Prelip M, Flores R, Kinsler J, Stevenson AM, Simonsen SE, Sharif M (2012). Evaluation of a statewide public health nursing training in Utah. Public Health Nurs.

[24] Bjerre LM, Paterson NR, McGowan J, Hogg W, Campbell CM, Viner G (2013). What do primary care practitioners want to know? A content analysis of questions asked at the point of care. J Contin Educ Heal Prof.

[25] Ely JW, Osheroff JA, Gorman PN, Ebell MH, Chambliss ML, Pifer EA (2000). A taxonomy of generic clinical questions: classification study. BMJ.

[26] Brekelmans G, Maassen S, Poell RF, Weststrate J, Geurdes E (2016). Factors influencing nurse participation in continuing professional development activities: survey results from the Netherlands. Nurse Educ Today.

[27] García García M, Valenzuela Mújica MP, Martínez Ocaña JC, Otero López M del S, Ponz Clemente E, López Alba T (2011). Results of a coordination and shared clinical information programme between primary care and nephrology. Nefrología.

[28] Lau R, Stevenson F, Ong BN, Dziedzic K, Treweek S, Eldridge S (2015). Achieving change in primary care effectiveness of strategies for improving implementation of complex interventions: systematic review of reviews. BMJ Open.

[29] Baxter P, DiCenso A, Donald F, Martin-Misener R, Opsteen J, Chambers T (2013). Continuing education for primary health care nurse practitioners in Ontario, Canada. Nurse Educ Today.

[30] Berggren E, Strang P, Orrevall Y, Olin AÖ, Sandelowsky H, Törnkvist L (2016). Evaluation of ConPrim: a three-part model for continuing education in primary health care. Nurse Educ Today.

